# The risk of uveitis in patients with JIA receiving etanercept: the challenges of analysing real-world data

**DOI:** 10.1093/rheumatology/kez449

**Published:** 2019-10-12

**Authors:** Rebecca Davies, Diederik De Cock, Lianne Kearsley-Fleet, Taunton Southwood, Eileen Baildam, Michael W Beresford, Helen E Foster, Wendy Thomson, Athimalaipet V Ramanan, Kimme L Hyrich, for the British Society for Paediatric and Adolescent Rheumatology Etanercept Cohort Study (BSPAR-ETN) and the Biologics for Children with Rheumatic Diseases (BCRD) study

**Affiliations:** 1 Arthritis Research UK Centre for Epidemiology, Centre for Musculoskeletal Research, The University of Manchester, Manchester Academic Health Science Centre, Manchester; 2 Institute of Child Health, University of Birmingham & Birmingham Children’s Hospital, Birmingham; 3 Clinical Academic Department of Paediatric Rheumatology, Alder Hey Children’s NHS Foundation Trust, Liverpool; 4 University of Liverpool and Alder Hey Children’s NHS Foundation Trust, Members of Liverpool Health Partners, Liverpool; 5 Musculoskeletal Research Group, Institute of Cellular Medicine, Newcastle University, Newcastle upon Tyne; 6 Paediatric Rheumatology, Great North Children’s Hospital, Newcastle upon Tyne; 7 Arthritis Research UK Centre for Genetics and Genomics, Centre for Musculoskeletal Research, Faculty of Biologic, Medicine and Health, The University of Manchester, Manchester; 8 National Institute of Health Research Manchester Biomedical Research Centre, Manchester University NHS Foundation Trust, Manchester Academic Health Science Centre, Manchester; 9 Bristol Medical School, University of Bristol, Bristol; 10 Paediatric Rheumatology, University Hospitals Bristol NHS Foundation Trust, Bristol, UK

**Keywords:** uveitis, JIA, biologic therapy, bias

## Abstract

**Objectives:**

To describe and compare the occurrence of newly diagnosed uveitis in children with JIA receiving MTX, etanercept, adalimumab and infliximab.

**Methods:**

This on-drug analysis included patients within UK JIA registries (British Society for Paediatric and Adolescent Rheumatology Etanercept Cohort Study and Biologics for Children with Rheumatic Diseases) with non-systemic disease, registered at MTX or biologic start with no history of uveitis. Follow-up began from date of first treatment, continuing until first uveitis, discontinuation of registered drug, most recent follow-up up or death, whichever came first. Hazard ratios comparing risk of uveitis between drugs were calculated using propensity-adjusted Cox regression.

**Results:**

A total of 2294 patients were included (943 MTX, 304 adalimumab/infliximab, 1047 etanercept). There were 44 reported cases of uveitis (27 MTX, 16 etanercept, 1 adalimumab). Unadjusted hazard ratio showed a reduced risk of uveitis in biologic cohorts compared with MTX. After adjusting for propensity deciles, there was no significant difference in the risk of uveitis between patients receiving etanercept or MTX [hazard ratio 0.5 (0.2–1.1)]. Fully adjusted comparisons were not possible for adalimumab/infliximab as there were too few events.

**Conclusions:**

In this first paper to compare the rate of new onset uveitis across the three main anti-TNF therapies used in JIA, a new diagnosis of uveitis is less common among patients starting biologics compared with MTX, although this did not reach statistical significance. The suggested protective effect of etanercept is likely explained by confounding, whereby patients in the MTX cohort are younger and earlier in disease, and therefore at greater risk of developing uveitis compared with etanercept patients.

**Rheumatology key messages**

- This is the first study comparing uveitis risk within each of the anti-TNF therapies.
- New diagnosis of uveitis is less common in JIA patients starting etanercept compared with MTX.
- The suggested protective effect of etanercept is likely explained by the influence of age and disease duration.

## Introduction

JIA is the most common inflammatory rheumatic disease in childhood, thought to affect around 4 in 1000 children [1, 2]. Uveitis is a significant comorbidity associated with JIA, with prevalence reported between 12 and 30% [3]. It is characterized by inflammation of the middle layer of the eye, and can result in significant visual morbidity [4], suggesting that diagnoses and treatment should be a priority.

There are a number of widely accepted risk factors for the development of JIA-associated uveitis, which include younger age at onset of JIA (<7 years) and the presence of ANAs [3]. There have also been reports of uveitis occurring more frequently in patients with oligoarticular JIA compared with polyarticular JIA, and in female patients [5, 6]. Uveitis is also thought to occur early in the JIA disease course, with a German study reporting that 73% of 406 patients who developed uveitis did so within the first year following JIA diagnosis [6].

In addition to these demographic and clinical factors, etanercept, a common TNF inhibitor treatment for JIA, has been considered as a potential risk factor in the development of uveitis in a cohort of patients with RA [7]. Within JIA, there has been a concern that etanercept can increase the likelihood of recurrence of uveitis in patients with a pre-existing history of the disease [1, 8], with one study suggesting that this was more common when patients were receiving etanercept monotherapy compared with etanercept and MTX in combination [9]. A randomized controlled trial exploring the role of etanercept as a treatment for uveitis found no difference in outcome between etanercept and placebo-treated patients [10], suggesting that among children with uveitis, etanercept is not an effective treatment, unlike other drugs within the TNF inhibitor class, including adalimumab via a randomized controlled trial (SYCAMORE) [11] and infliximab through a case series [12]. Whether or not etanercept is associated with an increased risk of developing new onset uveitis in patients with JIA is less clear. Similarly, although adalimumab and infliximab are effective treatments for many children with uveitis it is not known whether use of these drugs can prevent the onset of uveitis.

The aims of the present study are therefore to describe and compare the occurrence of newly diagnosed uveitis in children with JIA receiving MTX, etanercept, adalimumab and infliximab who do not have a history of uveitis at the start of therapy.

## Methods

### Patients

Patients recruited to one of two UK JIA national prospective treatment registries were included [the British Society for Paediatric and Adolescent Rheumatology Etanercept Cohort Study (BSPAR-ETN) established in 2004 and the Biologics for Children with Rheumatic Diseases (BCRD) study established in 2010]. To be enrolled in the studies, patients were required to have a diagnosis of JIA, classified according to the ILAR criteria [13]. A detailed explanation of the study methods of both studies has been described previously [14]. Patients starting etanercept (BSPAR-ETN) or a non-etanercept biologic (BCRD) for JIA are approached to join the respective studies alongside children starting MTX, who form a comparison cohort within the studies. Recruitment is recommended but not mandatory. Data are captured in identical manners regardless of which drug is started and which study the child is enrolled in. Both studies received ethical approval from a National Health Service Ethics Committee and written informed consent from parents (and where appropriate patients) was provided in accordance with the Declaration of Helsinki.

### Data collection and follow-up

Baseline data (defined as at the time of starting a biologic or MTX) were collected by the paediatric rheumatologist or clinical research nurse using a web-based questionnaire. Data collected include demographics (age, gender), disease status [disease duration, active joint count, limited joint count, ESR, CRP, physician global assessment, patient/parent global assessment, pain visual analogue scale, Childhood Health Assessment Questionnaire (CHAQ) [15], juvenile arthritis disease activity score-71] [16], ILAR disease classification, drug history and comorbidities. It is also recorded whether or not the patient has a history of uveitis at study registration and whether it was active at the time of registered drug start. ANA status was not collected over the period of patient recruitment included in this analysis. Follow-up data were extracted from the medical record at 6 months, 12 months and annually thereafter, and included current treatments and changes to anti-rheumatic therapy, as well as occurrence of serious and non-serious adverse events, including uveitis.

All adverse events are reported verbatim by the hospitals and coded centrally using the Medical Dictionary for Regulatory Activities [17].

Newly diagnosed uveitis cases were defined as any reported adverse event of uveitis in patients that had no previous history of uveitis recorded at baseline. Events coded to the Medical Dictionary for Regulatory Activities preferred terms ‘uveitis’ or ‘iridocyclitis’ (known as anterior uveitis) were included in the analysis. Additional information using a standardized proforma, including location and type of uveitis, was requested in all cases to verify the event as a new case of uveitis (*vs* a flare). Only first diagnoses of uveitis were included in our analysis.

### Statistical analysis

The analysis included all children with non-systemic JIA registered at the point of starting MTX, etanercept, adalimumab or infliximab who did not have a history of uveitis at the start of the registered drug. For the purpose of analysis, children starting adalimumab and infliximab were combined as numbers in each individual drug cohort were small. For all patients, person-years of exposure began from date of first treatment with the respective drug and continued until first diagnosis of uveitis, most recent study follow-up recorded up to 30 June 2018, discontinuation of registered drug or death, whichever came first. Events were only included if patients were receiving their treatment of interest (biologic or MTX) at the time of or within the 90 days preceding their first diagnosis of uveitis, to allow for any lag effect.

Patients who registered on MTX and later switched to etanercept or another biologic were followed in the MTX cohort until the point of biologic start. At this point they were censored from the MTX cohort and subsequently followed in the etanercept or adalimumab/infliximab cohort as described above. Similarly, patients who switched between biologics were followed in one cohort until the point of switch, from which point they were censored from the first cohort and followed up in the second cohort.

Baseline comparisons between cohorts are shown, using non-parametric descriptive statistics. Crude rates of uveitis are presented per 100 person-years with 95% CIs. Cox proportional hazard models were used to compare rates of newly diagnosed uveitis between the MTX and etanercept cohorts across all exposure time. Due to a lack of events, only unadjusted hazard ratios (HRs) are presented comparing MTX and adalimumab/infliximab or between etanercept and adalimumab/infliximab. Sensitivity analyses included (i) patients diagnosed with JIA under the age of 12 years (all follow-up included) and (ii) patients diagnosed with JIA under the age of 12 years with follow-up censored at 12th birthday. Current UK JIA uveitis screening guidelines suggest more frequent screening in patients younger than 12 years(3), therefore the latter two analyses were conducted to rule out any bias caused by this screening effect.

In order to reduce any effects of selection bias, a series of propensity scores stratified into deciles were used to adjust for potential confounding effects of baseline differences between the cohorts (etanercept *vs* MTX, etanercept-combination *vs* MTX, etanercept-monotherapy *vs* MTX and etanercept-combination *vs* etanercept-monotherapy) and included age, sex, disease severity (using baseline CHAQ and juvenile arthritis disease activity score-71), disease duration, baseline oral steroid use, ethnicity (white *vs* non-white) and ILAR category (Supplementary Table S1, available at *Rheumatology* online). The reported bias between the cohorts was low at between 1.5 and 5%. Two time-varying covariates were also included to estimate the probability of an etanercept-combination patient becoming an etanercept-monotherapy patient, and an etanercept-monotherapy patient becoming an etanercept-combination patient. These were included as covariates in the etanercept-combination *vs* etanercept-monotherapy model. Finally, a series of univariable Cox regressions were performed on baseline variables to identify possible risk factors in the development of new onset uveitis within the whole cohort.

All analyses were performed using Stata, version 14 (StataCorp, 2015, Stata Statistical Software: Release 14, College Station, TX, USA: StataCorp LP). Missing data were accounted for using multiple imputation (20 imputations), using the ice package in Stata [18]. As well as including baseline co-variates in the imputation model, uveitis incidence (quantified as whether a patient ever developed new onset uveitis) and log time to first uveitis were also included.

## Results

A total of 2698 patients with non-systemic JIA were recruited at point of starting one of the study drugs (1038 MTX, 540 adalimumab/infliximab, 1120 etanercept). Of these, 95 (9%), 236 (44%) and 73 (7%), respectively, had a history of uveitis at registration and were excluded from further analysis, resulting in a total of 2294 patients in the analysis; 943 MTX, 1047 etanercept and 304 adalimumab/infliximab. Patients in the final adalimumab/infliximab cohort consisted of 243 (80%) starting adalimumab and 61 (20%) starting infliximab. Baseline characteristics are presented in Table 1. The cohorts were relatively similar with respect to age and gender, but patients starting MTX were slightly younger and had much shorter disease duration compared with those starting biologics (median 2 years for both etanercept and adalimumab/infliximab *vs* 0 years for MTX). Patients starting MTX were more likely to have persistent oligoarthritis and patients starting adalimumab or infliximab were more likely to have enthesitis-related arthritis.

**Table 1:** Baseline characteristics of the etanercept, adalimumab, infliximab and MTX registered patients

Characteristic	MTX cohort	ETN cohort	ADA/INF cohort	Total missing data, *n* (%)
*N*	943	1047	304	—
Age, median (IQR), years	10 (4–13)	11 (6–14)	10 (6–13)	0
Gender, *n* (%)				
Female	662 (70)	721 (69)	197 (65)	0
Ethnicity, *n* (%)				
White	788 (84)	890 (86)	274 (90)	29 (1)
Disease duration, median (IQR), years	0 (0–1)	2 (1–5)	2 (1–5)	49 (2)
ILAR classification, *n* (%)				82 (4)
Oligoarthritis: persistent	160 (17)	55 (5)	16 (5)	
Oligoarthritis: extended	149 (16)	205 (20)	48 (16)	
Polyarthritis: RF-negative	330 (36)	400 (39)	107 (35)	
Polyarthritis: RF-positive	81 (9)	122 (12)	39 (13)	
PsA	82 (9)	75 (7)	25 (8)	
Enthesitis-related arthritis	72 (8)	101 (10)	62 (21)	
Undifferentiated arthritis	34 (4)	48 (5)	1 (1)	
Active joint count, median (IQR)	5 (2–9)	5 (2–9)	3 (1–6)	183 (8)
Limited joint count, median (IQR)	3 (1–7)	4 (1–8)	2 (1–6)	236 (10)
CHAQ score, median (IQR) 0–3	0.9 (0.3–1.5)	1.0 (0.3–1.6)	0.8 (0.3–1.4)	815 (36)
Pain VAS, median (IQR) 10 cm	5 (2–7)	5 (2–7)	4 (1.3–6.4)	787 (34)
ESR, median (IQR), mm/h	15 (7–30)	11 (5–25)	9 (5–22)	406 (18)
CRP, median (IQR), mg/l	5 (4–14)	5 (4–14)	5 (2–7)	385 (17)
Physician global assessment, median (IQR), 10 cm	4 (2–6)	4 (2–5)	3 (2–5)	803 (35)
Patient/parent global assessment, median (IQR), 10 cm	4 (2–6)	4 (2–6)	4 (1–6)	733 (32)
JADAS-71, median (IQR)	14 (9–23)	14 (8–20)	12 (7–18)	1233 (54)
Concurrent oral steroid use, *n* (%)	202 (21)	181 (17)	52 (17)	0
Concurrent MTX use, *n* (%)	—	555 (53)	202 (66)	0
Previous biologic exposure, *n* (%)	—	14 (1)	132 (43)	0

ADA: adalimumab; INF: infliximab; ETN: etanercept; IQR: interquartile range; CHAQ: childhood Health Assessment Questionnaire; VAS: visual analogue scale; JADAS-71: 71-joint juvenile arthritis disease activity score.

### Risk of new onset uveitis

There were 44 new diagnoses of uveitis over a total of 5456 person-years of follow-up: 27 in patients on MTX, 16 in patients on etanercept (etanercept-combination = 11, etanercept-monotherapy = 5) and 1 in a patient on adalimumab (Table 2). The majority of cases were unilateral at diagnosis with most children being diagnosed with anterior uveitis. There were no cases of panuveitis reported within this study. Cases were seen most frequently in those patients with oligoarticular or RF-negative polyarthritis (Table 3). Crude incidence rates, presented per 100 person-years, were 1.6 (95% CI 1.0, 2.3) in patients taking MTX, 0.6 (95% CI 0.3, 0.9) in those receiving etanercept and 0.1 (95% CI 0, 0.4) in patients receiving adalimumab or infliximab. The incidence rate was higher in patients in the etanercept-combination when compared with etanercept-monotherapy cohort.

**Table 2:** Crude incidence rates and HRs of new onset uveitis in patients on etanercept or adalimumab/infliximab *vs* MTX

	MTX	ETN	ADA/INF	ETN monotherapy	ETN–MTX combination therapy
Person-years of exposure	1701	2826	929	1707	1120
New diagnosis of uveitis, *n*	27	16	1	5	11
Crude incidence rates of uveitis (per 100 person-years)	1.6 (1.0–2.3)	0.6 (0.3–0.9)	0.1 (0–0.4)	0.3 (0.1–0.7)	1.0 (0.5–1.8)
Time from JIA diagnosis to uveitis diagnosis, median (IQR), years	2 (1–3)	4 (2–5)	2	4 (4–5)	4 (2–5)
Age at uveitis diagnosis, median (IQR), years	4 (3–9)	7 (6–10)	>15	7 (6.5–7.5)	9 (6–10)
Unadjusted HR of uveitis diagnosis (95% CI)*	Ref	0.4 (0.2, 0.7)	0.07 (0.009, 0.5)	0.2 (0.08, 0.6)	0.6 (0.3, 1.3)
	—	Ref	0.2 (0.02, 1.4)	—	—
PS-adjusted HR of uveitis diagnosis (95% CI)^a^*	Ref	0.5 (0.2, 1.1)	—	0.3 (0.08, 1.0)	0.6 (0.3, 1.6)
	—	—	—	Ref	2.6 (0.8, 8.8)
PS-adjusted HR of uveitis diagnosis (95% CI)^a^^,^^b^*	Ref	0.5 (0.2, 1.1)	—	0.3 (0.07, 0.9)	0.7 (0.3, 1.7)
	—	—	—	Ref	2.7 (0.8, 8.9)
PS-adjusted HR of uveitis diagnosis (95% CI)^a^^,^^c^*	Ref	0.6 (0.2, 1.4)	—	0.4 (0.1, 1.4)	0.7 (0.3, 1.7)
	—	—	—	Ref	2.4 (0.7, 8.1)

a Fully adjusted using propensity deciles (includes age, gender, CHAQ, JADAS, disease duration, ethnicity, comorbidity, baseline steroid use and ILAR category).

b Sensitivity analysis limited to patients younger than 12 years at JIA onset.

c Sensitivity analysis limited to patients censored at their 12th birthday.

* First line if all TNF drugs compared to MTX, second line is ETN compared to ADA/INF. ADA: adalimumab; INF: infliximab; ETN: etanercept; IQR: interquartile range; HR: hazard ratio; PS: propensity decile; CHAQ: childhood Health Assessment Questionnaire; JADAS: juvenile arthritis disease activity score.

**Table 3:** Characteristics of new onset uveitis cases in patients on etanercept or adalimumab/infliximab *vs* MTX

	MTX	ETN	ADA/INF	ETN monotherapy	ETN–MTX combination therapy
New diagnosis of uveitis, *n*	27	16	1	5	11
New diagnosis of uveitis by ILAR subtype, *n*					
Oligoarthritis: persistent	6	1	0	1	0
Oligoarthritis: extended	5	9	0	3	6
Polyarthritis: RF-negative	13	4	1	0	4
Polyarthritis: RF-positive	1	0	0	0	0
PsA	0	1	0	0	1
Enthesitis-related arthritis	1	1	0	1	0
Undifferentiated arthritis	1	0	0	0	0
Uveitis location, *n* (%)					
Bilateral	9 (33)	6 (38)	0	0	6 (55)
Unilateral	14 (52)	6 (38)	1 (100)	4 (80)	2 (18)
Not stated	4 (15)	4 (24)	0	1 (20)	3 (27)
Uveitis type, *n* (%)					
Anterior	19 (70)	9 (56)	1 (100)	5 (100)	4 (36)
Panuveitis	0	0	0	0	0
Not stated	8 (30)	7 (44)	0	0	7 (64)

ADA: adalimumab; INF: infliximab; ETN: etanercept.

The mean age at uveitis diagnosis was 7 years in the etanercept cohort *vs* 4 years in the MTX cohort, with time from JIA diagnosis to uveitis onset 4 and 2 years, respectively. The adalimumab patient was over 15 years of age at the time of uveitis diagnosis and this occurred 2 years post-JIA diagnosis. Unadjusted HR showed a reduced risk of uveitis in all etanercept cohorts and the adalimumab/infliximab cohort when compared with patients on MTX; however, after adjusting for propensity deciles, there was no significant difference in the risk of uveitis between patients receiving etanercept or MTX [HR 0.5 (95% CI 0.2, 1.1)]. Although the rates were higher in patients receiving etanercept in combination with MTX compared with those receiving it as monotherapy, this did not reach statistical significance [HR 2.6 (95% CI 0.8, 8.8)].

Similar results were found in sensitivity analysis limited to children with a JIA diagnosis before the 12th birthday and in an analysis censored at the child’s 12th birthday (Table 2).

### Risk factors in the development of newly diagnosed uveitis

A univariable analysis of risk factors in the development of new uveitis (Table 4) found a significant association between development of uveitis and younger age at baseline, shorter disease duration, being of non-white ethnicity and having oligoarticular disease (compared with other ILAR categories excluding RF-negative polyarthritis). Gender, disease severity and functional disability were not found to be significantly associated with development of uveitis in this cohort.

**Table 4:** Univariable predictors of new onset uveitis in all patients [presented as HR (95% CI)]

Predictor	New onset uveitis [HR (95% CI)]
Gender	
Male	Ref
Female	1.7 (0.9, 3.1)
Age at baseline (years)	0.8 (0.7, 0.8)
Disease duration at baseline (years)	0.9 (0.8, 0.9)
Ethnicity	
Non-white	Ref
White	0.5 (0.3, 0.8)
ILAR subtype	
Other subtype	Ref
Oligoarthritis	2.5 (1.3, 4.9)
RF-negative polyarthritis	1.4 (0.7, 2.9)
Oral CS use at baseline	1.3 (0.7, 2.3)
CHAQ at baseline	1.2 (0.9, 1.7)
JADAS-71 at baseline	1.0 (0.9, 1.0)

HR: hazard ratio; CHAQ: childhood Health Assessment Questionnaire; JADAS-71: 71-joint juvenile arthritis disease activity score.

## Discussion

Uveitis is accepted to be one of the most common complications of JIA, with a variety of recognized risk factors such as younger age and oligoarticular subtype [3]. There is concern that etanercept may flare disease in those with pre-existing uveitis [1, 8]. As a consequence, etanercept is rarely the first choice of biologic in patients with JIA with a history of uveitis. However, the relationship between etanercept and the new development of uveitis in patients with JIA remains unclear.

From this analysis, which has used data from children and young people enrolled in national cohort studies of treatments for JIA, no association was found between the use of etanercept and the occurrence of new uveitis when compared with those receiving MTX for the first time, although the crude incident rates were lower in patients receiving etanercept. Concurrent MTX use with etanercept did not appear to have a further protective effect in this cohort. Given the low occurrence of events in the adalimumab/infliximab cohort, it is difficult to conclude whether these drugs had any protective effect over the occurrence of uveitis and the data should not be used to preferentially treat children with no history of uveitis with one treatment over another.

The lower rates of uveitis among patients starting etanercept do not support a causative link between etanercept and the development of uveitis. However, it should be noted that the patients in the MTX and etanercept cohorts differed significantly with respect to their baseline risk of uveitis. Patients starting MTX did so early in disease course and were also more likely to have oligoarthritis. Subsequently, they also developed their uveitis at a younger age (median 4 *vs* 7 years). Younger age (<7 years) is an accepted risk factor in the development of uveitis [5] and may explain in part the difference in crude rates observed between etanercept- and MTX-treated patients. Thus, by the time patients with JIA start etanercept, they may be inherently at a lower overall risk of developing uveitis, a so-called ‘healthy user’ effect, which may be more prominent in the ‘older’ etanercept cohort who are further along in their disease course, consistent with recent findings that uveitis is most common in the first year of disease [6]. It is also possible that a further selection bias occurred with regard to choice of first biologic in more recent years. As knowledge about a possible association between etanercept and uveitis became more widespread, patients who were perceived by their treating paediatric rheumatologist as having a higher risk of developing uveitis, such as those who were younger, ANA positive or had an oligoarticular subtype, might have been steered away from etanercept treatment towards an alternative biologic. Unfortunately information on ANA was not captured in this study at the time of recruitment of patients included in this analysis, but no significant different in age between patients starting adalimumab/infliximab or etanercept was observed.

The main strengths of the study are related to the size of the cohorts, with close to 2300 patients included in this study, the detailed follow-up procedures used and the prospective study design minimizing potential recall bias. Furthermore, extensive uveitis information is captured from centres using specially designed proformas, which ask for the type, localization and course of uveitis, as well as establishing whether it is a new or recurrent event. This minimizes the risk of events being misclassified as new if in fact the patient has had uveitis previously.

As a non-randomized observational treatment study, the study is subject to the limitations common to all such research. Despite the overall large sample size, the size of the cohort of children starting adalimumab or infliximab who did not already have uveitis was relatively low. There were also missing data across all covariates, although there was not a complete lack of information for any patient and therefore, multiple imputation was used to account for these missing covariate data.

In conclusion, this study found that a new diagnosis of uveitis is less common among patients with JIA starting etanercept compared with MTX, although this did not reach statistical significance. The suggested protective effect of etanercept is likely explained by the influence of age and disease duration, whereby patients in the MTX cohort are, on average, younger and so more ‘at risk’ of developing uveitis compared with etanercept patients. As a consequence, and in the absence of a sufficient comparison group, our understanding of what additional risk etanercept adds when looking at the risk of developing uveitis remains unclear.

*Funding*: The recruiting centres were supported by the National Institute for Health Research Clinical Research Network in England. This report includes independent research supported by the National Institute for Health Research Biomedical Research Centre. The views expressed in this publication are those of the author(s) and not necessarily those of the National Health Service, the National Institute for Health Research or the Department of Health. The authors also acknowledge the Arthritis Research UK Centre for Epidemiology (Grant No. 20380) for infrastructure and technical support in data collection and statistical analysis. The Biologics for Children with Rheumatic Diseases (BCRD) study is funded by Arthritis Research UK Grant 20747. The British Society for Paediatric and Adolescent Rheumatology Etanercept Cohort Study (BSPAR-ETN) study is funded by a research grant to the University of Manchester from the British Society for Rheumatology (BSR). The BSR has received restricted income from Pfizer. This income finances a wholly separate contract between the BSR and the University of Manchester, who provide and oversee the data collection, management and analysis of the data. The principal investigator and her team have full academic freedom and are able to work independently of pharmaceutical industry influence. All decisions concerning analyses, interpretation and publication are made autonomously of any industrial contribution. No specific additional funding was received from any bodies in the public, commercial or not-for-profit sectors to carry out the work described in this article.

*Disclosure statement*: A.R. has received honoraria/speaker fees/consultant from Abbvie, UCB, Eli Lilly, Novartis and SOBI; K.L.H. has received honoraria/speaker fees from Abbvie and BMS. Grants to the institution have been received from BMS, Pfizer and UCB. R.D., D.D.C., L.K.-F., T.S., E.B., M.W.B., H.E.F. and W.T. have no conflicts to declare.

## Supplementary Material

kez449_Supplementary_Data
